# Changes in intraocular pressure and ocular pulse amplitude of rhesus macaques after blue light scleral cross-linking

**DOI:** 10.1186/s12886-022-02306-0

**Published:** 2022-02-22

**Authors:** Chong Liu, Yu Li, Mengmeng Wang, Jing Li, Ningli Wang, Fengju Zhang

**Affiliations:** 1 Beijing Ophthalmology & Visual Sciences Key Lab, Beijing Tongren Eye Centre, Beijing Tongren Hospital, Capital Medical University, No. 1 Dongjiaomin Xiang, Beijing, 100730 Dongcheng District China; 2 Hebei Ophthalmology Key Lab, Hebei Provincial Eye Hospital, Xingtai, Hebei Province China

**Keywords:** Blue-light, Cross-linking, Rhesus macaques, Intraocular pressure, Ocular pulse amplitude

## Abstract

**Background:**

Scleral cross-linking can enhance the biomechanical strength of the sclera and is expected to be a new operative method for the prevention of myopia. However, studies investigating the changes in intraocular pressure (IOP) and ocular pulse amplitude (OPA) after blue light-riboflavin induced scleral collagen cross-linking (SXL) in rhesus monkeys are limited. This study aimed to investigate the changes in IOP and OPA in three-year-old rhesus macaques 1 week, 1 month, and 3 months after blue light-riboflavin SXL.

**Methods:**

Seven three-year-old rhesus macaques (14 eyes) were randomly divided into two groups, with 4 monkeys in group A (8 eyes) and 3 monkeys in group B (6 eyes). The right eye of each rhesus macaque was used as the experimental eye, whereas the left eye was used as the control. In group A, one quadrant of each right eye was irradiated. In group B, two quadrants of each right eye and one quadrant of each left eye were irradiated. The IOP and OPA of both eyes were measured in all seven rhesus macaques before SXL and 1 week, 1 month, and 3 months postoperatively, and differences in the IOP and OPA between the experimental and control eyes were evaluated via the paired t test.

**Results:**

In groups A and B, there were no significant differences between the experimental and control eyes in the IOP or OPA before SXL or 1 week, 1 month, or 3 months postoperatively (*P* > 0.05).

**Conclusions:**

The IOP and OPA are not significantly affected in 1 vs 0 or in 1 vs 2 quadrants of blue light SXL.

## Background

Myopia is an ocular disease affecting public health globally. It is estimated that by 2050, almost half the world’s population will have myopia, and one-tenth of the world’s population will develop high myopia [1]. Myopia, especially high myopia, primarily manifests as scleral thinning and progressive growth of the ocular axis; hence, methods that enhance the biomechanical strength of the sclera with a resultant delay in scleral thinning and axial growth can effectively prevent and treat myopia and its complications. Scleral cross-linking (SXL) with riboflavin and blue light has a stiffening effect on the sclera in rabbits [2]. The biomechanical strength of the human sclera may be enhanced by collagen cross-linking with riboflavin/460-nm blue-light irradiation [3]. Wang M et al. confirmed that the scleral biomechanics after equatorial SXL are stronger than those after posterior SXL and that the equatorial region of the sclera is the preferred position for SXL [4]. Anatomically, the vortex veins pass through the equatorial sclera, and any abnormality in the vortex veins can induce a pathological change in the intraocular pressure (IOP) and ocular blood flow. The ocular pulse amplitude (OPA) refers to the difference in the IOP between the systolic and diastolic periods of the cardiac cycle and is a parameter reflecting the blood flow in the eyes. IOP fluctuations are indicative of the rhythmic haemodynamic status of the eye corresponding to each heartbeat. There have been many reports confirming that patients with diseases associated with changes in the blood flow in the eye have relatively low OPA values. Abeg. O Pinto L et al. found that the OPA value in patients with primary open-angle glaucoma and normal-IOP glaucoma was lower than that in healthy people of the same age [5]. Wang et al. proved that there was no significant difference in the OPA between the two eyes in patients with bilateral disease but that there was a significant difference in patients with unilateral disease [6]. Ebru Nevin Çetin et al. confirmed that the OPA in patients with Behcet's disease with ocular lesions was lower than that in patients with Behcet's disease without ocular lesions and that in the normal controls [7]. Ebru n. Cetin et al. also confirmed that the OPA in patients with multiple sclerosis was lower than that in the controls [8]. Based on the experiences of our research group from a previous investigation, this study aimed to observe the changes in the IOP and OPA after blue light-riboflavin induced SXL in the eyes of rhesus monkeys and provide some experimental basis for the clinical application of the blue light SXL technique.

## Methods

### Study design

**Fig. 1:**
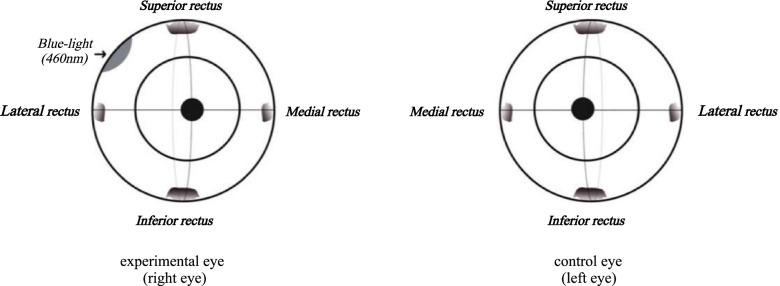
Front views of rhesus macaque eyeballs showing the scleral collagen cross-linking locations in group A

**Fig. 2:**
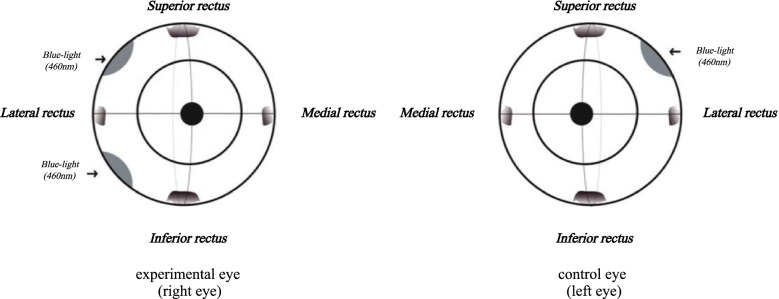
Front views of rhesus macaque eyeballs showing the scleral collagen cross-linking locations in group B

Seven three-year-old rhesus macaques (14 eyes) were divided into two groups using a completely randomized design, with 4 monkeys in group A (8 eyes) and 3 monkeys in group B (6 eyes). The right eye of each rhesus macaque was used for the experiment, and the left eye was used as a control. In group A, the superior temporal quadrant of the right eye was irradiated with blue light (Fig. 1). In group B, the superior and inferior temporal quadrants of the right eye and the superior temporal quadrant of the left eye were irradiated with blue light (Fig. 2). The estimation of the sample size in each group for this study was performed based on other reports in the literature [9–11]. Both eyes of all the rhesus macaques were examined clinically before SXL and 1 week, 1 month, and 3 months postoperatively, and the examiners were blinded to the group allocation during the experiment.

### Subjects

Seven healthy rhesus macaques (age, 3.0–3.5 years; weight, 4.3–6.0 kg) were obtained from the Experimental Animal Centre of the People’s Liberation Army Academy of Military Medical Sciences in China. They were raised in the laboratory animal breeding room of Capital Medical University. They were housed individually and provided free access to food and water throughout the study. The room temperature was maintained between 23 °C and 27 °C, and the relative humidity was maintained between 45 and 55%. The general condition of all macaques met the national health monitoring standards for primate laboratory animals. They underwent anterior-segment and fundus examinations before initiation of the experimental procedure to exclude potential health factors that might affect the IOP measurements. They showed no abnormities, and baseline measurements of the IOP showed no pathological findings. The use of experimental animals in this study was approved by the Institutional Animal Care and Use Committee of Capital Medical University (AEEI-2014–127), and all macaques included in the study were acquired and used in compliance with the Association for Research in Vision and Ophthalmology’s Statement for the Use of Animals in Ophthalmic and Vision Research. After the experiment, the rhesus macaques continued to receive good care and were used in subsequent studies.

### Surgery and SXL

All macaques were anaesthetized with a mixture of ketamine hydrochloride (10%; 20 mg/kg) and xylazine hydrochloride (5 mg/kg) injected intramuscularly into the buttocks. All surgical procedures were performed by one surgeon (Mengmeng Wang). All eyes underwent a 360-degree conjunctival peritomy, and Tenon’s capsule was dissected to expose the equatorial sclera in the corresponding quadrant. Two scleral traction wires were placed in front of the planned cross-linking site; these wires were pulled to rotate the eyeball and expose the irradiation zone. A 0.5% solution of riboflavin was instilled on the exposed scleral surface 20 min before irradiation. The diameter of the irradiation zone in the equatorial sclera in all animals was 10 mm. A collimated beam of blue light (460 nm) was generated with a beam illumination system (Beijing Obodi Optoelectronic Technology Co., Ltd., Beijing, China), which conformed to the national standards. The exposed sclera was irradiated from a distance of 5 cm, with a resulting surface area of irradiation of 22.5 mW/cm2; riboflavin solution was dripped onto the scleral surface every 20 min during irradiation. Blank irradiation zones were not irradiated, but the 0.5% riboflavin solution was administered in the same manner. Postoperatively, the preset sutures were removed, and the conjunctiva was sutured with absorbable surgical sutures. A 0.3% gatifloxacin eye gel was applied to all eyes to avoid infection, 4 times a day for 1 week.

### IOP Measurements

The IOP measurements were performed before SXL and 1 week, 1 month, and 3 months after the SXL operation. After the induction of general anaesthesia, each macaque was secured in a seated position. An appropriately experienced examiner then measured the IOP of the experimental and control eyes in groups A and B using a Model 30 pneumatonometer (Reichert, America) and TonoLab rebound tonometer (iCare Finland Oy, Helsinki, Finland). When performing measurements using the Model 30 pneumatonometer, the operator held the probe and moved the sensor towards the eye gently to touch the corneal apex. As the membrane touched the cornea, the sensor handle was continuously moved towards the eye until an audible tone was generated, which indicated that the most accurate reading was obtained. The tone changed to a noticeably low pitch when the standard deviation among IOP readings was maintained below 1.0 mmHg for three seconds. Additionally, the instrument displayed the average IOP and standard deviation. When performing measurements using the TonoLab rebound tonometer, the IOP was assessed 5 times, and for each of these assessments, it was measured 6 times, and the mean IOP values were calculated.

### Measurement of OPA

The OPA measurements were performed before SXL and 1 week, 1 month, and 3 months after the SXL operation. After the induction of general anaesthesia, the rhesus monkeys were placed in a sitting position. An appropriately experienced examiner then measured the OPA of the experimental and control eyes in groups A and B using the Model 30 pneumatonometer. When measuring the OPA in pulse tonometry mode, the operator held the probe in contact with the cornea. A changed tone was generated when the pneumatonometer sensed five ocular pulses, indicating that the instrument had enough samples to compute an average OPA; the test ended when the instrument sensed ten ocular pulses. The reading displayed was the average of all the detected pulses. The measurement was repeated 3 times, and the mean value of the 3 measurements was obtained.

### Statistical Analysis

SPSS version 20.0 (SPSS, Chicago, IL, USA) was used to analyse the results. The IOP and OPA data are recorded as the mean ± standard deviation. The Shapiro–Wilk test was used to test normality. We evaluated differences in the IOP and OPA between the experimental and control eyes via the paired t test at the scheduled follow-up time points within both groups. *P* < 0.05 was considered statistically significant.

## Results

Seven three-year-old rhesus macaques (14 eyes) were included and randomly divided into two groups: group A (8 eyes) and group B (6 eyes). Complete 3-month follow-up data were obtained for all eyes. Postoperatively, all macaques had normal corneas, anterior chambers, and clear lenses, and none exhibited any vitreous or retinal lesions. Moreover, there were no clinical signs of inflammation after blue light SXL.

**Fig. 3:**
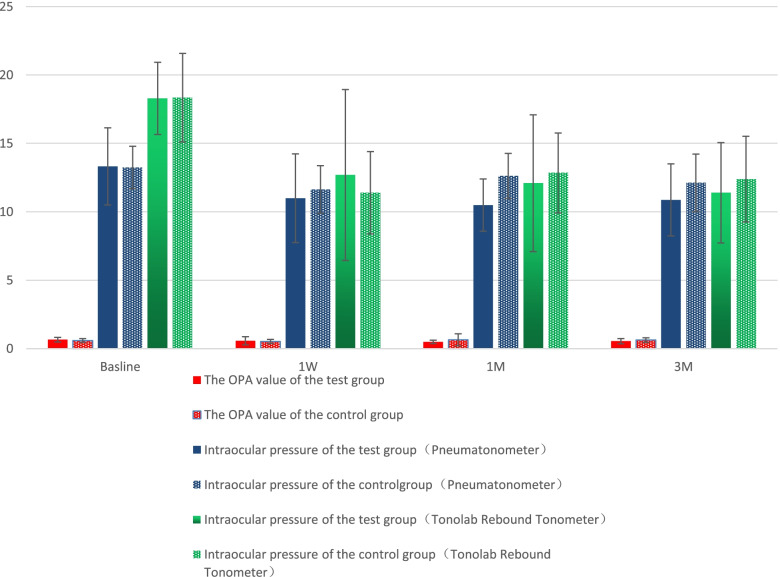
IOP and OPA measurements in the experimental and control eyes in group A. IOP: Intraocular pressure; OPA: Ocular pulse amplitude

The baseline OPA in the seven rhesus monkeys (14 eyes) was 0.65 ± 0.06 mmHg. The IOP and OPA measured by the TonoLab rebound tonometer/pneumatonometer and the pneumatonometer before SXL and 1 week, 1 month, and 3 months postoperatively showed no significant differences between the experimental and control eyes in group A (*P* > 0.05; Fig. 3, Table 1).

**Table 1:** Demographics and characteristics of the study animals at baseline

Animal#	Sex	Weight(kg)	IOP(mmHg)		OPA(mmHg)	
			R	L	R	L
1	M	5.6	15.2	14.4	0.65	0.5
2	M	4.9	19.2	20.8	0.5	0.5
3	F	6.0	21.4	21.2	0.6	0.6
4	F	5.4	17.4	17.0	0.9	0.8
5	M	4.3	19.4	21.6	0.8	0.3
6	F	5.2	13.6	12.4	1.1	0.9
7	F	4.8	13.0	14.0	0.5	0.5

**Fig. 4:**
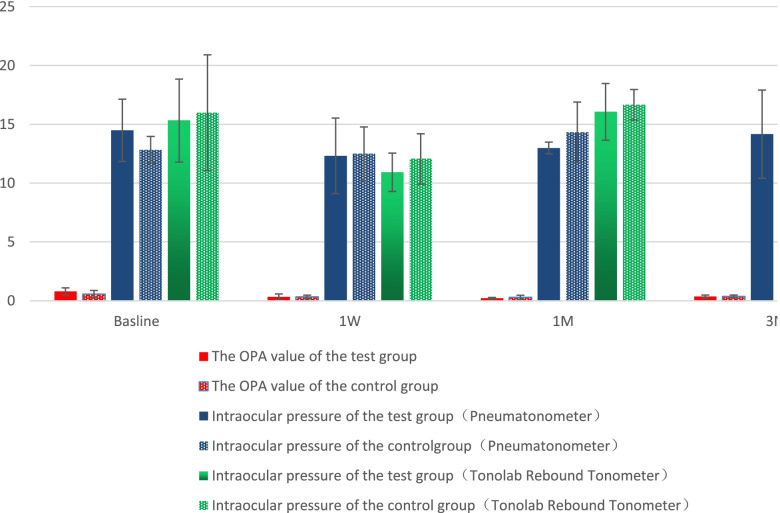
IOP and OPA measurements in the experimental and control eyes in group B. IOP: Intraocular pressure; OPA: Ocular pulse amplitude

Additionally, the IOP and OPA measured by the TonoLab rebound tonometer/pneumatonometer and the Model 30 pneumatonometer, respectively, before SXL, and 1 week, 1 month, and 3 months postoperatively showed no significant differences between the experimental and control eyes in group B (*P* > 0.05; Fig. 4, Table 2). However, there was a significant difference in the IOP measured by the pneumatonometer and TonoLab rebound tonometer. Moreover, the values obtained by the pneumatonometer were significantly lower than those obtained by the TonoLab tonometer (t = -2.671, *P* = 0.010).

**Table 2:** IOP and OPA measurements in the experimental and control eyes in two groups

		IOP_a_(mmHg)			IOP_b_(mmHg)			OPA(mmHg)		
		E eye	C eye	*P* value	E eye	C eye	*P* value	E eye	C eye	*P* value
Group A	Base	18.30 ± 2.64	18.35 ± 3.24	0.931	13.33 ± 2.82	13.25 ± 1.55	0.919	0.66 ± 0.17	0.60 ± 0.14	0.194
	1w	12.70 ± 6.25	11.40 ± 3.01	0.556	11.00 ± 3.24	11.63 ± 1.75	0.600	0.58 ± 0.30	0.53 ± 0.15	0.718
	1 m	12.10 ± 5.00	12.85 ± 2.92	0.582	10.50 ± 1.91	12.63 ± 1.65	0.110	0.50 ± 0.12	0.65 ± 0.44	0.519
	3 m	11.40 ± 3.67	12.40 ± 3.13	0.239	11.88 ± 2.63	12.13 ± 2.10	0.495	0.55 ± 0.19	0.63 ± 0.17	0.391
Group B	Base	15.33 ± 3.53	16.00 ± 4.92	0.572	14.50 ± 2.65	12.83 ± 1.15	0.199	0.80 ± 0.30	0.57 ± 0.31	0.250
	1w	10.93 ± 1.63	12.07 ± 2.14	0.360	12.33 ± 3.21	12.50 ± 2.29	0.995	0.33 ± 0.25	0.33 ± 0.15	1.000
	1 m	16.07 ± 2.41	16.67 ± 1.30	0.449	13.00 ± 0.50	14.33 ± 2.57	0.448	0.23 ± 0.06	0.30 ± 0.17	0.423
	3 m	13.80 ± 2.03	13.27 ± 2.10	0.094	14.17 ± 3.75	14.33 ± 2.75	0.874	0.37 ± 0.12	0.37 ± 0.12	0.423

*IOP*_*a*_ Intraocular pressure (Tonolab Rebound Tonometer), *IOP*_*b*_ Intraocular pressure (Pneumatonometer), *OPA* Ocular pulse amplitude, *E eye* experimental eye, *C eye* control eye, *Base* baseline, *w* week, *m* month

## Discussion

The progression of myopia is primarily manifests as scleral thinning and progressive growth of the ocular axis. The use of SXL to increase scleral rigidity and reduce axial eye elongation has been suggested, and most of the current related research has been based on collagen cross-linking (CXL) by riboflavin/ultraviolet –A light. Compared to ultraviolet light, blue light is more controllable because of its longer wavelength. Blue light-riboflavin induced SXL was found to increase the biomechanical strength of the sclera in rabbits and donated human eyes [2, 3]. Wang M et al. confirmed that the scleral biomechanics were stronger after equatorial ultraviolet- and riboflavin-induced SXL than after posterior ultraviolet riboflavin SXL [4]. Moreover, equatorial SXL prevents damage to the optic nerve; hence, the equatorial region of the sclera is the preferred location for SXL [4]. Our research group conducted a series of studies on equatorial SXL, Yu Li et al. [12] irradiated one quadrant in their study on the safety and long-term scleral biomechanical stability of rhesus eyes after scleral cross-linking with blue light irradiation; Yu Li et al. [13] irradiated one or two quadrants with blue light for riboflavin scleral cross-linking in an ocular safety evaluation. Anatomically, the vortex veins pass through the equatorial sclera. They transport uveal blood outside the eyeball and maintain the outflow of partial aqueous fluid or humour; therefore, any abnormality in the vortex veins can induce a pathological change in the IOP and ocular blood flow. At present, the OPA is mostly analyzed in the studies of glaucoma and other ocular ischemic diseases, but it has not been used to study SXL. In addition, compared with other experimental animals, monkeys have eyes that are more structurally similar to human eyes physiologically. This study is the first to investigate the OPA in rhesus macaques and has more clinical significance than studies performed in other experimental animals.

The OPA refers to the difference in IOP between the systolic and diastolic periods of the cardiac cycle and is indicative of the rhythmic haemodynamic status of the eye corresponding to each heartbeat. The mechanism of change in the OPA is as follows: during the systolic period of the cardiac cycle, blood pumped from the heart enters the blood vessels of the eye, of which 90–95% enters the choroidal blood vessels. OPA values in normal individuals fluctuate between 2 and 3 mmHg but are usually maintained at less than 5 mmHg; moreover, binocular differences are often less than 0.5 mmHg [14–17]. In this study, the baseline OPA in the rhesus macaques was 0.65 ± 0.06 mmHg, which is lower than previously reported values between 2 and 3 mmHg. This difference can be attributed to the influence of general anaesthesia on OPA measurements. Pianka et al. reported that both sub-Tenon's and peribulbar anaesthesia can significantly reduce the OPA [18]. Zuche et al. reported significantly reduced IOP and OPA values under general anaesthesia [19]. However, this difference could also be due to the species-specific differences between rhesus monkeys and humans.

The Model 30 pneumatonometer is a novel, noninvasive pneumatic tonometer; its probe contains a gentle, floating pneumatic sensor that touches the surface of the cornea with the exact amount of applanating force required to take a tonometry measurement. In this experiment, there were significant differences in the IOP measured by the Model 30 pneumatonometer and the TonoLab rebound tonometer; the values obtained by the Model 30 pneumatonometer were significantly lower than those obtained by the TonoLab tonometer (*P* < 0.05). Barkana et al. found that the IOP values measured by the Model 30 pneumatonometer in the seated and supine positions (both measured when the individual was awake) were higher than those measured by the iCare rebound tonometer [20]. Therefore, the differences in this study might be related to general anaesthesia. The difference between the two tonometers may be associated with the rapid fluctuations in IOP after the induction of general anaesthesia. Van der Walt et al. found that the IOP decreased 8 min after the administration of an intramuscular ketamine injection [21]. Ding et al. found that in animal experiments, the IOP could increase rapidly and subsequently decrease quickly to a lower level within 3 min after the induction of general anaesthesia by ketamine [22]. The relatively small sample size may also be one of the reasons for this difference.

Gool et al. measured the OPA in normal individuals and glaucoma patients and reported that the OPA and IOP values were positively correlated; for every 10 mmHg increase in the IOP, the OPA increased by 1.6 mmHg [23]. Hence, OPA values should be compared on the basis of similar IOP values. In this study, the IOP values in both groups A and B showed no significant difference between the experimental and control eyes. Therefore, the comparison of the OPA values in this experiment is of clinical significance. In this study, there were no significant differences between the experimental and the control eyes in the IOP or OPA before SXL or 1 week, 1 month, or 3 months postoperatively in group A or group B (*P* > 0.05). This result indicats that the IOP and OPA in rhesus macaques were not significantly affected by blue light SXL in one or two quadrants. However, the results could also indicate that SXL has a limited therapeutic scope and may not adequately affect the blood flow in the entire eyeball. Scleral crosslinking may increase ocular rigidity (OR). OR is an index to quantify the compliance of the globe and has been used to characterize the biomechanical properties of the ocular wall. Friedenwald [24] uses the OR coefficient (E) in a logarithmic equation to express the pressure–volume relationship: E = (log IOP 1 − log IOP 2)/(V1 − V2). In the formula, the slope E is the OR coefficient. Wang proposed that the OPA and pulsatile ocular blood flow could be used to approximately replace the (log IOP 1 − log IOP 2) and (V1 − V2) values in the above formula to obtain an estimated value E (Er) [25]. A higher OPA may be found in eyes with increased rigidity, due to either a higher rigidity coefficient or a higher IOP [26]. Girard MJ et al. considered that changes in the scleral properties detected in eyes of monkeys subjected to elevated IOP include stiffening, and these changes are believed to reflect remodelling of the scleral extracellular matrix [27]. Therefore, OPA may not be directly related to simple ocular rigidity, but ocular rigidity and OPA value change owing to the increase in intraocular pressure. Kimball and Elizabeth considered scleral crosslinking capable of increasing the risk of glaucoma; however, we found that the increase in IOP was induced in a separate step. It was not a result of the stiffening. Local change in ocular rigidity is unlikely to affect global ocular rigidity. This is also documented in the following publication, where a substantial part of the corneal stromal thickness was excised and the overall rigidity did not change significantly [28]. Moreover, the postoperative OPA values in both the experimental and control eyes were lower than the baseline values in group B, which may be because the surgical intervention results in a decrease in the OPA. Therefore, we can include other detection indices of ocular blood flow in future experiments to further understand the changes in blood flow. However, the lack of a significant difference between the two eyes in the two groups provides a basis for the safety of the surgery. In addition, some limitations of the study should be noted. First, the results are credible based on the sample size reported in other studies in the literature, but the sample size in this investigation was still small, and we plan to expand the sample size in further research. Second, the OPA measurements indicate the blood flow in the entire eyeball and not the blood flow only in the irradiated area. However, other equipment that can measure local blood flow cannot obtain IOP values in a short time. Third, although the IOP and OPA were measured according to the manufacturers' instructions and we took the average values of multiple measurements, both the IOP and OPA are variable values, and the data may be affected by fluctuation.

## Conclusion

In conclusion, the IOP and OPA are not significantly affected in 1 vs 0 or in 1 vs 2 quadrants of blue light SXL. This study provides an experimental basis for the clinical application of the blue light SXL technique. Further studies incorporating scleral biomechanical sclera stability will be performed to obtain a relatively optimized schema of SXL.

## Data Availability

The datasets used and/or analyzed during the current study are available from the corresponding author on reasonable request.

## Abbreviations

IOP: Intraocular pressure
OPA: Ocular pulse amplitude
CXL: Collagen crosslinking
SXL: Scleral collagen crosslinking
OR: Ocular rigidity
